# “*We get support now* …”: a mixed methods study of patients’ experiences of healthcare under the national health insurance scheme (PM-JAY) in India

**DOI:** 10.1186/s12913-025-13632-6

**Published:** 2025-11-28

**Authors:** Swati Srivastava, Divya Parmar, Christoph Strupat, Viktoria Couturier, Susanne Ziegler, Sharmishtha Basu, Manuela De Allegri

**Affiliations:** 1 Heidelberg Institute of Global Health, Medical Faculty and University Hospital, Heidelberg University, 69120 Heidelberg, Germany; 2 Department of Population Health Sciences, School of Life Course and Population Sciences, King’s College London, London, SE5 9RJ UK; 3 Economic and Social Systems, German Institute of Development and Sustainability (IDOS), 53113 Bonn, Germany; 4 Deutsche Gesellschaft für Internationale Zusammenarbeit (GIZ), Friedrich-Ebert-Allee 32, Bonn, Germany; 5 Indo-German Social Security Programme (IGSSP), Deutsche Gesellschaft für Internationale Zusammenarbeit (GIZ) GmbH, New Delhi, 11002 India

**Keywords:** Patient experience, Publicly-funded health insurance, India

## Abstract

**Background:**

Publicly-funded health insurance (PFHI) schemes are widely employed in low- and middle-income countries to enhance financial protection and advance universal health coverage. India’s Pradhan Mantri Jan Arogya Yojana (PM-JAY), launched in 2018, provides inpatient coverage to over 500 million socioeconomically disadvantaged individuals. Although positive patient experiences are linked to improved health outcomes, evidence on patient experiences under PM-JAY remains limited. This mixed-methods study investigates patient experiences with PM-JAY healthcare services, while incorporating provider reflections on these experiences.

**Methods:**

A concurrent triangulation mixed-methods study was conducted across 16 districts in 7 Indian states. Qualitative data were collected via semi-structured interviews (*n* = 219 doctors, *n* = 55 beneficiaries) and 28 focus group discussions with beneficiaries. Quantitative data included 508 patient exit surveys, 115 hospital surveys, and 115 infrastructure checklists. Quantitative data were analyzed using descriptive statistics, while qualitative data were analyzed using content analysis. Data triangulation occurred during the analysis phase.

**Results:**

While hospitals had the required physical amenities and patient exit surveys indicated very high satisfaction with PM-JAY services, the qualitative interviews and group discussions with beneficiaries and healthcare providers revealed several areas needing procedural and service improvements. Chief among these were the need for better communication, and enhanced abilities of PM-JAY implementers to provide empathetic and coordinated care. There were also stark differences across states: beneficiaries in Chhattisgarh, Gujarat, Kerala, Meghalaya and Tamil Nadu were more appreciative of PM-JAY services, while those in Bihar and Uttar Pradesh reported significant dissatisfaction.

**Conclusions:**

Beneficiaries and healthcare providers identified key areas for improvement, including patient-provider communication, empathy, emotional support, care coordination, and quality. Barriers included limited awareness of scheme processes among beneficiaries and providers, and deficient communication skills. Addressing organizational and structural challenges, alongside using informal community networks via frontline health workers, offer opportunities to enhance PM-JAY healthcare delivery.

**Supplementary Information:**

The online version contains supplementary material available at 10.1186/s12913-025-13632-6.

## Background

Universal health coverage (UHC) encompassing access to the full range of quality healthcare services without financial hardship for all persons, is a critical target under the Sustainable Development Goals [1]. Publicly-funded health insurance (PFHI) is being extensively used to advance progress toward UHC by increasing financial protection for healthcare utilization in low- and middle-income countries (LMICs) [2]. Dignity, compassion and respect for patients and the provision of healthcare that meets patients’ needs and expectations are essential components of quality healthcare under UHC [3–5], including for healthcare delivered through PFHI.

Patient experience of healthcare comprises patients’ encounters and reports of both medical (e.g., technical) and non-medical (e.g., interpersonal) aspects of clinical care, and includes the full range of patient encounters with the health system [3, 6]. Positive patient experiences are closely associated with improved outcomes for patients and healthcare providers. These benefits include increased adherence to prevention and treatment protocols, better clinical outcomes, improved patient safety in hospitals and increased use of healthcare services [7–9]. Additionally, patient experiences can provide valuable feedback to service providers, helping them to be more responsive and aligned to patients’ needs and expectations [9, 10].

Aspects of patients’ experience of service use under different PFHI schemes include experiences with different processes of receiving care, such as waiting time, shared decision making by patients and healthcare providers, satisfaction and trust [11]. However, limited attention has been paid to understanding these issues in LMICs [11–13]. A systematic review of publications on Nigeria’s national scheme found long waiting times leading to patient dissatisfaction and unfavorable experiences [14]. Another study from Nigeria found that patients placed great value on autonomy, communication and prompt attention [15]. A study on Indonesia’s PFHI scheme found that while patients gave high ratings for the ease of understanding healthcare providers, they expressed dissatisfaction with long waiting times [16]. Long waiting times, lack of cleanliness and limited clinical examinations during consultations adversely affected patient experiences in a study on Mexico’s social security scheme [17].

Further, healthcare providers’ perspectives on their capacities to meet patients’ expectations has been examined even less in the context of health insurance reforms [11, 18, 19]. From a methodological perspective, most of these studies are either quantitative assessments [16, 17], or are systematic or scoping reviews covering some aspects of patient-centered care [13, 14, 18, 19]. Qualitative studies focusing on patients experience of healthcare under PFHI in LMICs lens are scarce.

### Patient experiences of healthcare within the Pradhan Mantri Jan Arogya Yojana

The *Pradhan Mantri Jan Arogya Yojana* (PM-JAY), a national PFHI, was implemented in India in 2018 for more than 500 million socially and economically disadvantaged Indians. Beneficiaries are entitled to select inpatient services in empaneled public and private hospitals with an annual coverage amount of INR 500,000 (approximately USD 5850 in 2025) per household. This includes 3-day pre-hospitalization and 15-day post-hospitalization expenses, covering preventive and rehabilitative services, diagnostics and medicines. Beneficiaries are required to verify their PM-JAY eligibility by a patient navigator, known as *Ayushman Mitra*, prior to hospitalization. Ayushman Mitras coordinate the necessary scheme-related processes, documentation and information exchange for patients and hospitals, from the point of admission to discharge [20]. PM-JAY empanels all secondary level and higher public hospitals, while private hospitals have to meet certain basic empanelment criteria for physical amenities such as physical infrastructure, human resources including doctors and nurses, bed strength; and further specialized criteria based on the specific benefit package of services for which hospitals are empaneled. Staff within hospitals such as the treating physicians, nurses and other ancillary staff should be oriented to scheme processes and requirements, in order to facilitate implementation [21].

There is limited understanding of how patients are experiencing services under PM-JAY. A study from six Indian states found low awareness among beneficiaries about the scheme and their entitlements, which can adversely affect their health-seeking behavior and healthcare utilization [22]. A study in two Indian states found that prompt attention to patients’ needs, easy access to information about the scheme, and patient satisfaction with services provided were rated significantly better in public versus private hospitals by patients, however 26% of insured patients still incurred out-of-pocket expenditures (OOPE) [23]. Another study from the same two states found that the majority of patients incurred OOPE, and that hospital staff and Ayushman Mitras were trained on technical elements, but not on PM-JAY processes related to dealing with and assisting patients [24]. An earlier study in the state of Chhattisgarh reported frequent formal and informal payments, and harsh, asymmetrical interactions between patients and healthcare providers under the predecessor scheme of PM-JAY, the Rashtriya Swasthya Bima Yojana or RSBY [25]. Only two of these studies used qualitative methods, eliciting information on patient experiences by asking healthcare providers about patient experiences of healthcare utilization under PM-JAY [21, 24], and no study interviewed patients directly.

Evidence on patient experiences of care outside of PFHI in India is also scarce. A study on patient experiences of care by older adults from 2017 to 18 found that most individuals reported “good” treatment in six domains of waiting time, respectful treatment, clarity of explanations provided to them, privacy during consultation, treated by provider of choice, and cleanliness of facility; however, negative experiences were higher for public facilities, particularly for waiting time and facility cleanliness [26]. Two other small-scale studies exploring patient experiences among cancer patients [27] and hospitalized patients [28] also found high self-reported satisfaction with health care provider interaction and communication. The available evidence indicates that systematic evidence on patient experiences of healthcare in India is limited.

This mixed-methods study aims to understand how patients experience receiving healthcare under PM-JAY, while also incorporating healthcare providers reflections on these patient experiences.

## Methods

### Study design, data collection and setting

We conducted a concurrent triangulation mixed methods study, in which qualitative and quantitative data were collected in parallel. We used multiple tools to collect qualitative (semi-structured interviews) and quantitative (patient exit surveys, a hospital survey, and infrastructure checklist) data. Triangulation of the two data streams took place during analysis. The study was embedded within a larger demand- and supply-side evaluation of the PM-JAY [29].

The states of the study sample, namely Bihar, Chhattisgarh, Gujarat, Kerala, Meghalaya, Tamil Nadu and Uttar Pradesh, were purposively selected through consultations between study funders and national PM-JAY implementers to reflect the variation in PM-JAY implementation models (those operated by a trust, an insurance company or a mix of both, please refer to Supplementary Table 1 for more information) as well as states’ prior experiences with implementing PFHI (Table 1). Within each state, we purposively selected two districts, representing a well- and an average-performing district with regards to implementation. These districts were selected as below-average performing districts did not have an adequate number of PM-JAY empaneled hospitals providing services since the commencement of the scheme, which would make identification of respondents who had used services difficult. More details are available elsewhere [29]. Within districts, we sampled different sets of respondents for different data collection approaches, as outlined below.

**Table 1:** Study data collection approaches and sample sizes

	Quantitative data collection		Qualitative data collection		
State	Hospital Survey and Infrastructure Checklist (n)	Patient Exit Survey (n)	Administrator and Medical Doctor Interviews (n)	Beneficiary Focus Group Discussions (n)	In-depth Interviews (n)
Bihar	12	22	24	4	8
Chhattisgarh	19	97	37	4	8
Gujarat	21	108	40	4	9
Kerala	13	98	26	4	8
Meghalaya	20	81	38	4	8
Tamil Nadu	13	74	24	4	8
Uttar Pradesh	17	28	30	4	8
**Total**	**115**	**508**	**219**	**28**	**55**

All data was collected between November 2019-March 2020. This period constituted the initial implementation period [30] after the roll-out of PM-JAY, when scheme processes were being operationalized, adjusted and adapted in the different states, and therefore our findings are indicative of this phase of early uptake.

### Quantitative data

#### Hospital survey and infrastructure checklist

For this study, we used data from 115 PM-JAY empaneled hospitals sampled in the umbrella PM-JAY evaluation study (Table 1) [29]. The sampling strategy was designed to purposively sample 6 empaneled and 6 non-empaneled hospitals per district; however, many districts had more than 6 empaneled hospitals. In order to obtain more information on the status of the supply-side implementation in these districts, more empaneled hospitals (which were willing to participate) were sampled by replacing non-empaneled hospitals, hence our sample includes at least 6 hospitals per district. Within each hospital, we administered a quantitative infrastructure checklist to hospital administrators to collect information on hospital amenities and facilities critical for PM-JAY empanelment, and a survey to hospital directors on their perceptions with PM-JAY implementation. Indicators on hospital amenities and facilities were based on essential empanelment criteria for the scheme, and hence were used to assess the extent to which empaneled hospitals were fulfilling these criteria.

#### Patient exit survey

We further administered a quantitative survey to all exiting PM-JAY patients being discharged from hospitals on the day of data collection. These surveys collected information on patients’ experiences with receiving care under PM-JAY, and related OOPE. Under PM-JAY guidelines, patients should not have had to incur any OOPE for services under the scheme. We conducted a total of 508 patient surveys (Table 1). 28 sampled hospitals had no PM-JAY patients being discharged on the day survey teams visited. All PM-JAY patients (100%) being discharged on the day of the visit in the remaining hospitals consented to and were administered the patient exit survey. The mean age of patients was 44 years and there were equal males and females.

These three quantitative tools were digitized and administered through computer-assisted personal interviewing using tablets. Questions from all quantitative tools used for this study are available as supplementary file 1.

### Qualitative data

#### Hospital semi-structured interviews

We conducted semi-structured interviews with hospital directors or, in their absence, with senior hospital administrators, as well as with a senior physician in each of the 115 hospitals. These interviews collected information on administrative, clinical and patient care-related experiences of hospital staff involved with service provision under PM-JAY. We conducted a total of 209 interviews. These interviews lasted on average 39 minutes, with about 80% of the respondents being male and an average age of 45 years.

These interviews were conducted by trained interviewers from IQVIA Consulting and Information Services India Pvt. Ltd.

#### Beneficiary focus group discussions

In addition to hospital-level data, we also collected data from PM-JAY eligible persons and persons who had utilized services through PM-JAY in communities. We purposively selected two communities (one in an urban and one in a rural setting) from areas with high numbers of PM-JAY eligible persons within each district (Table 1). Local PM-JAY and primary healthcare staff assisted in identifying PM-JAY eligible adults. We organized a total of 28 Focus Group Discussions (FGDs, 4 in each state, 2 per district, Table 1), with 8–15 adult participants in each FGD; these lasted for 60 minutes on average. To ensure that gender dynamics did not influence the discussions, FGDs consisted either of all female or all male participants. We conducted 15 all-female and 13 all-male FGDs.

#### Beneficiary in-depth interviews

From each FGD, we identified two critical information-rich cases, i.e., individuals whose experience with PM-JAY service use (either for themselves or for an immediate family member for whom they were a caregiver) appeared to be particularly relevant to our objective. We conducted a total of 55 In-depth Interviews (IDIs, approximately 8 per district) with information-rich individuals (Table 1); these lasted for 60 minutes on average. A total of 26 IDIs were with women and the remaining with men.

FGDs and IDIs explored knowledge and awareness about the scheme, healthcare utilization under PM-JAY, including perceptions of quality of care and experiences with healthcare providers. Trained interviewers from Nielsen India Pvt. Ltd collected this data. Discussion guides used for the qualitative data collection are available as supplementary file 2.

There was no respondent overlap between the beneficiary qualitative and hospital quantitative and qualitative data collection. Data were collected in the state’s local language or English. All participants provided written informed consent. Data collectors worked under close supervision of the study team with bi-weekly debriefings [31]. Interviews were transcribed verbatim and translated into English by the interviewers. The full sample description is given in Table 1.

### Analytical approach

The analytical approach followed a mixed deductive-inductive strategy [32]. We identified the initial themes relating to person/patient-centered and responsive care as mentioned in literature [33–36]. These included, empathy, respect, engagement, relationship, communication, shared decision-making, holistic focus, individualized focus and coordinated care, as well as patients’ expectations and experiences of care. Three coders then read through a subset of beneficiary FGD and IDI transcripts (approximately 10 transcripts each) to identify initial codes based on the themes identified above. These codes were discussed among the authors and adapted to develop an interim coding frame. Next, indicators in the quantitative tools were then mapped to this coding frame, and descriptive statistics generated. All quantitative analysis was conducted using Stata version 14.

**Fig. 1:**
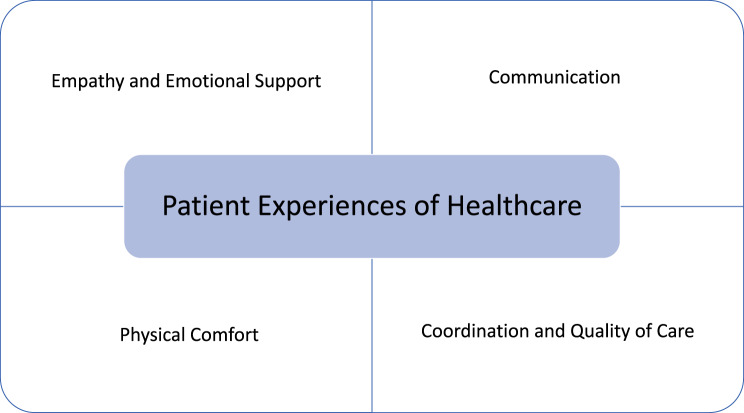
Themes on patient experiences

Then, a group of three coders coded the beneficiary qualitative data, and a group of two coders coded the hospital qualitative data. The coding process in both of these groups entailed regular discussions to redefine and adapt codes and interpret material, and joint review of each other’s coded material within the groups. The analysis was conducted using a combination of coding in NVivo until saturation [37, 38] was attained for each state, followed by preparing manual summaries of the coded material for each respondent group, by state. Next, the set of codes developed by both beneficiary and hospital coding groups were jointly contrasted and compared to arrive at condensed themes and jointly interpret and appraise all qualitative data. This process led to the identification of the following themes on patient experiences: themes of **empathy** (respect for patient preferences) and **emotional support** (including involvement of family), **communication** (information, education and communication), **physical comfort** and **coordination and quality of care** (access to and continuity of care) (refer to Fig. 1), as critical for respondents in the processes of utilizing and experiencing healthcare [39]. Summaries of the joint interpretation by themes were also prepared.

From the qualitative data, we observed that OOPE were a critical factor affecting patient experiences, and we included this code under the theme coordination of care. The available quantitative data was again mapped to these final themes. For the infrastructure checklist data, we identified and mapped indicators on infrastructure availability to the respective themes of empathy and emotional support, communication, physical comfort and coordination of care. For the patient exit data, we identified and mapped indicators to the themes of patient satisfaction and coordination of care.

Finally, thematic summaries of the qualitative data and quantitative results were jointly interpreted, contrasted and compared to arrive at the results.

## Results

We present the triangulated findings across data streams for each of the themes in the analytical approach (Fig. 1). Under the coordination and quality of care theme, we present findings to reflect the sequence of different PM-JAY processes beneficiaries undergo when accessing care. We differentiate the respondents of the exit survey (referred to as patients) from the qualitative inquiry at community level (referred to as beneficiaries) in order to better contextualize the results. Some themes draw more from qualitative information, while others are more reliant on quantitative information. The names of districts are not presented to maintain confidentiality.

### Empathy and emotional support

Beneficiaries across states reported instances of kind, attentive and respectful, as well disrespectful and impolite interactions with doctors, nurses and hospital functionaries. In most states, being assisted by frontline health workers or local government authorities eased the admission process and increased attentiveness of hospital staff. Further, beneficiaries perceived the PM-JAY card (i.e., proof of benefit entitlements under the scheme) as empowering and that hospital staff were more responsive to their needs: “*After this card, you can keep 2–4 rupees in your pocket and you can visit hospital. We get support now*.” – Female Beneficiary, FGD, Chhattisgarh.

Especially in Bihar, beneficiaries stated that they were only respectfully spoken to once accompanied by frontline health workers during admission. These more disrespectful interactions were often attributed to beneficiaries’ appearance of lower socioeconomic status: “*There was no one willing to listen … Those who will be big men there they only listen to them … Either there be an officer or staff, only they listen them, nobody will listen to the common man … If a poor man goes to [hospital name] then no one is going to listen to him …. There is no one who will listen to a poor man.*” – Male Beneficiary, IDI, Bihar.

“*Their response to each person will change - like if they are a farmer, their response will be different than if a person wears pant shirt. Response will be different. If he was a politician then they will respond differently. But they must not be like that, they must treat everyone equally. They can’t treat well only if the person is educated*.”— Male Beneficiary, IDI, Tamil Nadu.

There were also several reports of care provided without explicit patient consent (such as: a female beneficiary undergoing a non-consented surgery in Uttar Pradesh), violations of patient privacy (such as: a female beneficiary whose privacy was not maintained post-operatively and who had to ask for curtains to be drawn), or denial of attendants to be present for social support after visiting hours: “*One person must be there to take care of the patient when they were serious, right? One person must be there. But they [hospital staff] won’t allow us. At night 12 o’clock they will send us outside [of the ward]* …” – Male Beneficiary, IDI, Tamil Nadu.

### Communication

Beneficiaries across states positively experienced communication with hospital staff. Beneficiaries in Bihar and Chhattisgarh noted that hospital staff communication efforts improved towards PM-JAY patients, and staff come across as more accepting of them. Some beneficiaries noted differences in communication styles with respect to doctors who often had limited time to interact with them, compared to nurses or other hospital staff who were able to invest more time in interpersonal interactions. Conversely, some beneficiaries in Bihar reported that doctors’ behavior changed for the worse once they learned that the patient was availing services under PM-JAY, and this deterred them from asking questions or interacting with hospital staff regarding the scheme. Sometimes, hospital staff used intimidation to prevent beneficiaries from asking questions: “*They say, “What do you want to know?” in very bad tone. So, we get scared and do not understand where to go*?” – Female Beneficiary, FGD, Bihar.

Beneficiaries also remarked that as PM-JAY patients, staff took more time to discuss and explain procedures to them in more detail. Doctors in Meghalaya explained that extra funds available to hospitals via PM-JAY allowed different priority setting in healthcare provision, which also improved the providers’ work morale: “*Like I said upgradation, total upgrade. Patient was getting everything within record time, great turnaround time, but the most important thing I have seen is, the best thing in fact, is the change in behavior among the healthcare providers*.” – Hospital Director, Public Hospital, Meghalaya.

Sometimes this greater focus on PM-JAY patients seemed motivated by the fear of repercussions as PM-JAY established stronger patient complaint procedures. As a result, hospital staff took more time ensuring beneficiaries were sufficiently familiar with and aware of patient entitlements under PM-JAY. Hospital staff also noticed that they themselves were not always properly trained in patient communication: “*I don’t know if there is any training that has been arranged for the persons who are doing that job of uploading the records and patient record maintenance and communication to the patient. I am not aware of any training that has been done for the people who are doing that job. So, if that can be done on a regular basis, they will also understand the problems and it would be a combined approach*.” – Hospital Administrator, Public Hospital, Meghalaya.

A lack of understanding regarding PM-JAY entitlements or benefits appears to be linked to shortages of Ayushman Mitras, i.e., dedicated staff whose task it is to communicate PM-JAY-related processes to beneficiaries. Often, there were no Ayushman Mitras on duty, and other staff members had to fill in this role in parts. “*There should be a separate staff for Ayushman Mitra because at present, there is too much workload on one data entry operator due to which he is often stressed out*.” – Medical doctor, Public Hospital, Bihar.

**Fig. 2:**
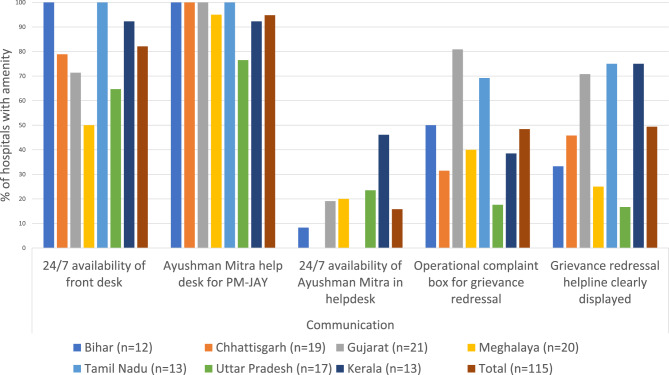
Communication and information services under PM-JAY

Our survey data shows that on average 2.2 Ayushman Mitras were employed per hospital, (Fig. 2) and approximately 82% of hospitals made PM-JAY front desks available 24/7 to attend to PM-JAY patients. Only 16% of hospital front desks (ranging from zero hospitals in Chhattisgarh and Tamil Nadu to 46% Kerala) had a 24/7 presence of an Ayushman Mitra staff to facilitate specific PM-JAY patient registration and admission processes (Fig. 2).

Beneficiaries often felt that they did not have sufficient information on PM-JAY processes or such information was not provided in a language they understood well enough to make informed decisions. Throughout the process of beneficiary verification, which was conducted at independent kiosks, primary healthcare facilities or at hospitals, beneficiaries often lacked clarity about the procedure and the reasons for the need for such thorough verification. In Gujarat and Meghalaya, for example, beneficiaries felt apprehensive about providing their signatures and thumbprints without understanding what they were supposed to sign off, but nevertheless did as they were told. Some were further concerned that any of their existing government benefits could be taken away once they provide their signature. Beneficiaries in Kerala who contacted the toll-free helpline to understand more about these verification processes, found that such information was only provided in Hindi but not in their local language.

“*When our pictures and all were taken, we were not told anything about this [signing documents], that yes or no … I’ll tell you that most here are not educated. So, when somebody says that fill this paper or form and you’ll get this. All the filling up work is done immediately by us …*” – Female Beneficiary, FGD, Gujarat.

Beneficiaries often lacked information on which hospitals provided services covered by PM-JAY. In many instances in Bihar and Uttar Pradesh, beneficiaries traveled considerable distances to hospitals, only to be refused services under PM-JAY. “*There is no use of it [PM-JAY card] … even if we go to a government owned hospital or any other hospital then they [hospital authorities] say that treatment can’t be done by this [PM-JAY] smart card*.” – Male Beneficiary, FGD, Chhattisgarh.

Conversely, reasons for denial of admission from hospital staff included a lack of clarity regarding the PM-JAY benefit package admission categories, and the inconsistencies around reimbursement rates.

Many beneficiaries would have preferred to receive more details on the care they received, such as diagnoses, treatments, or the benefit package under which they were admitted. Often, the amount that was deducted from their available PM-JAY coverage limit was not explicitly disclosed. Beneficiaries felt this information to be essential for better accountability, especially since some expenses were automatically charged to their PM-JAY accounts while others had to be paid out of pocket. Most beneficiaries complained that they were not aware prior to their admission that additional costs related to their care might have to be borne by them. Hence, full disclosure of the purpose of OOPE requested by hospitals prior to treatment initiation would have been useful.

Many beneficiaries also reported being denied services under PM-JAY because hospital staff were not aware of the specific details of the scheme: “… *But in this case [staff lack of awareness] where should we go for more information? … Then the hospital authorities said that if we ourselves don’t know how can we inform you*?” – Male Beneficiary, IDI, Bihar.

On the other hand, hospital respondents reported that beneficiaries had limited awareness of PM-JAY entitlements and processes. Hospital staff found beneficiaries to be more demanding of their rights to healthcare once they knew of their entitlements, and asked for additional treatments or services such as private rooms: “… *people come with mindset that they have ATM with them and they can take any services they desire*.” – Medical doctor, Private Hospital, Gujarat.

Both hospital administrators and doctors voiced a need to have formal communication processes between hospitals, PM-JAY administration, and beneficiaries: “*My only submission or input will be that on regular basis such interactions should happen with the third party [PM-JAY implementation agencies], with the beneficiaries and the hospital, so that, if at all there is some gap, any implementation gap, or any communication gap is there, that can be resolved*.” – Hospital director, Private Hospital, Tamil Nadu.

### Physical comfort

Most beneficiaries in almost all states reported that hospitals appeared clean and safe, with patients provided with food and water regularly. Beneficiaries perceived private hospitals provided better infrastructure services in Bihar, Uttar Pradesh and Chhattisgarh; whereas in Meghalaya, Kerala and Tamil Nadu they felt that public hospitals were better. However, a majority of beneficiaries in Bihar complained about the physical amenities: “*There was no bed, no food, not a single glass of water.*” – Male Beneficiary, IDI, Bihar.

**Fig. 3:**
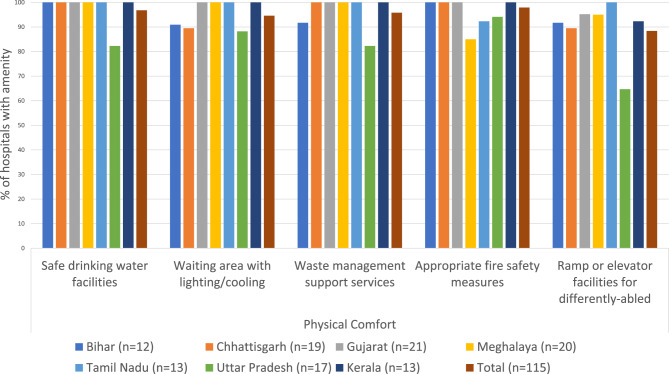
Physical comfort of patients under PM-JAY

Our survey data further shows that hospitals in all states were able to provide patient amenities, such as safe drinking water, comfortable waiting areas, appropriate waste management services and fire safety measures, except for ramps or elevators for the differently-abled which were available in 65% of hospitals in Uttar Pradesh (Fig. 3).

### Coordination and quality of care

**Fig. 4:**
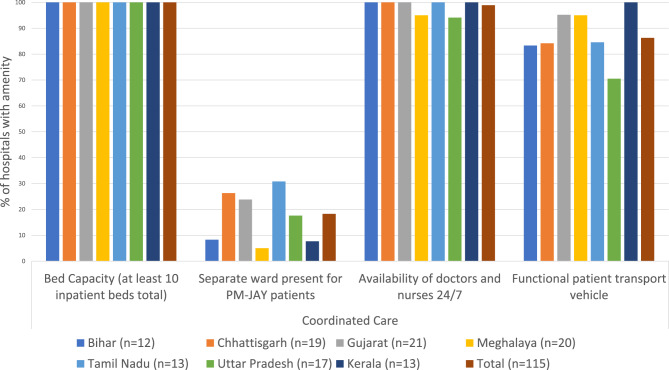
Coordination of care under PM-JAY

Our survey data showed that almost all hospitals had infrastructure to coordinate patient care within and between hospitals, including adequate bed capacities, 24/7 availability of doctors and nurses, and access to patient transport (Fig. 4). On average, hospitals had 9.5 (range 6.7–12.8) of available specialty services (out of 23 specialty services covered under PM-JAY) empaneled (Fig. 4).

Beneficiaries across states felt that the hospital attentiveness and services had improved because of PM-JAY. However, increases in the demand of hospital-based services after PM-JAY implementation led to increases in resource requirements (especially regarding maintenance of patient records and documentation) in some hospitals, especially in Chhattisgarh and Kerala: “*Scheme [patients under PM-JAY] patients require more staff nursing because of the high demand of patients compared to the free treatment patients[patients admitted as walk-in patients, not under PM-JAY]. At present, we are in need of staff members as we are low on them*.” – General Practitioner, Public Hospital, Tamil Nadu.

Some hospitals offered separate wards for PM-JAY patients (approximately 18% of hospitals, Fig. 4). Some beneficiaries perceived this separation based on insurance status to reflect a lower standard of care provided to PM-JAY patients. Our survey data shows that almost 27% of exiting patients were admitted to dedicated PM-JAY wards (Table 2).

**Table 2:** Responses on health service experience from exiting PM-JAY patients (%)

Indicator	Bihar	Chhattisgarh	Gujarat	Meghalaya	Tamil Nadu	Uttar Pradesh	Kerala	Total
Waiting time < 30 min	95.4	50.5	43.9	69.6	78.4	42.9	83.7	64.2
31–59 min	4.6	49.5	41.2	10.1	14.9	28.6	15.3	26.7
1–3 hours	0	0	14.0	20.3	6.7	28.5	1.0	8.9
> 3 hours	0	0	0.9	0	0	0	0	0.2
Patient admitted in separate PM-JAY ward	40.9	9.3	12.9	2.5	60.8	39.3	46.9	26.8
Incurred OOPE for pre-hospitalization	45.5	6.2	2.9	22.2	33.9	25.0	80.6	29.1
Referral to other PM-JAY facilities	4.6	2.1	7.6	2.6	9.5	3.6	2.0	4.6
Incurred OOPE during current hospitalization	13.6	2.1	0.9	17.9	21.6	21.4	70.4	22.1
**TOTAL (N)**	**22**	**97**	**108**	**81**	**74**	**28**	**98**	**508**

Interviewed medical doctors and administrators across states explained that beneficiaries and paying patients were provided the same medical care, but admitted that different types of medicines, supplies, and consumables had to be provided to PM-JAY beneficiaries to limit the treatment costs allotted by some of the PM-JAY package rates: “*In critically sick patients at times you think you need to provide the highest antibiotics but there’s no provision, there is no clause [in PM-JAY]. So, at times we feel frustrated while treating medical patients on the medical packages. Due to low package cost, the financial burden comes either on the patient or hospital has to bear it or compromise with the quality*.” – Medical doctor, Private Hospital, Chhattisgarh.

In our survey, 99% of exiting patients reported being satisfied (i.e., good, very good or excellent) with services they received under PM-JAY, and 98% would recommend the hospital to others. Similarly, 99% of patients rated their satisfaction with the PM-JAY scheme as good, very good or excellent. However, group discussions with beneficiaries portrayed a more mixed experience, with beneficiaries in Chhattisgarh, Gujarat, Kerala, Meghalaya and Tamil Nadu (on average) more satisfied with medical services received, while those in Bihar and Uttar Pradesh were dissatisfied because of long waiting times, the need for informal payments, poor interpersonal interactions and the lack of medicines and supplies, which led to OOPE (further elaborated below).

Almost all interviewed beneficiaries incurred expenditures prior to hospitalization for travel, initial consultations and diagnostic procedures, medicines and other medical supplies. Doctors in Kerala reported that delays in pre-authorization (approvals needed from PM-JAY implementation agencies to admit patients) resulted in patients either being denied treatment, or being forced to wait for up to a week without treatment; these affected patient experiences and patients also had to pay for some services in this period. Nearly 36% of exiting patients had to wait beyond 30 minutes to be first seen by a healthcare provider and about 29% of exiting patients incurred pre-hospitalization OOPE (i.e., incurred expenses for this hospitalization episode before being admitted in the hospital, Table 2). Some or all pre-admission expenses were reimbursed by hospitals to beneficiaries after discharge; this was particularly frequent in Meghalaya and Kerala. These reimbursements were often done due to fear of complaints: “*The change is that now, we have to return the money [spent on] the diagnosis of the outpatient or in OPD [out-patient department] of [by the] PM-JAY patient. We also have to return the money of the tests conducted of whatever amount like 700, 800, 1000 or 2000 [Rupees] because if someone complains, then we have to pay three times [as much] as fine. So, we give minimum tests to be conducted in diagnosis to avoid refund of more amounts*.” – Hospital Director, Private Hospital, Chhattisgarh.

A recurring complaint in Bihar and Tamil Nadu was the need for informal payments to hospital guards and administration for doctors or nurses to see patients. “*If we give 50 rupees to them [hospital guards], the things done in private hospital will also be done in a government hospital. To get a blood test result if they [hospital staff] ask us to come tomorrow then if I give money to them, I will get the report within 1 hour. Everything is only money in a government hospital*.” – Male Beneficiary, FGD, Tamil Nadu.

Beneficiaries also voiced differential treatment based on ability to pay, proximity to bureaucrats or local government, or other structural determinants: “*It requires money to get proper treatment from there [hospital] … once you pay in full, your treatment will be done right away. Those who have money, they get their treatment done first. Those who do not have money, they have to wait*.” – Male Beneficiary, FGD, Uttar Pradesh.

”*In [government district hospital] common people have no value. If they go, they face many problems. Those who have lobby and reach upper bureaucrats, he can avail the treatments well and can get medicines from the hospital. If you have no reach then you have to buy all medicines from outside, like everything from outside*.” – Male Beneficiary, FGD, Bihar.

Once admitted, beneficiaries again incurred OOPE for medicines and consumables, diagnostic procedures, and often food. About 22% of exiting patients incurred OOPE during hospitalization (Table 2). Purchasing medicines was reported to be a deterrent to continuing treatment by beneficiaries in Meghalaya. Sometimes, beneficiaries would be asked to purchase expensive medicines and supplies from outside, in both private and public hospitals: “*Brokerage is more. They [doctors] suggest the patients to buy medicines from their favorite medicine shops*.” – Male Beneficiary, FGD, Bihar.

Some beneficiaries reported being admitted in private rooms, without being informed these costs will not be covered by PM-JAY and therefore had to pay OOPE for room charges while their treatment was paid under PM-JAY. Beneficiaries undergoing longer-term treatments such as chemotherapy for cancer or for renal dialysis incurred substantial and repeated OOPE in several states, despite these conditions being covered under PM-JAY. As these conditions did not entail hospitalization but could be treated as ambulatory services, many hospitals across all states either did not cover them or asked patients to pay first and reimbursed them only when the hospital received the funds from PM-JAY. “*In my wife’s case as she is a cancer patient, she didn’t need to get hospitalized but needs continuous treatment. We need to spend everything first and then only get claims only if they [hospital staff] have proof that we spend firstly and then get reimbursed. She didn’t get the reimbursement of the last visit till now*.” – Male Beneficiary, IDI, Kerala.

However, some beneficiaries also reported that apart from travel expenses (which were partially subsidized in hospitals in Chhattisgarh, Gujarat and Kerala), the entire expenses for their admission were borne through PM-JAY: “*Getting benefit is easy. If we did not have the [PM-JAY] card then my husband’s eye would not be treated. I am poor, how would I pay 10000 or 15000 rupees*?” – Female Beneficiary, FGD, Uttar Pradesh.

Nearly 5% of exiting patients were referred to other facilities for different services during their hospitalization (Table 2). Beneficiaries reported being referred to higher-level public facilities or to private facilities from public hospitals, but this often meant paying for additional travel costs for long distances. Beneficiaries reported being denied care under PM-JAY at the private referral hospitals, because private hospitals preferred to admit them as paying patients.

Beneficiaries reported variable experiences with the discharge process. Depending on the indication for admission, post-operative devices and consumables—such as baby kits in Chhattisgarh or spectacles in Uttar Pradesh—were provided at discharge. Some beneficiaries reported being threatened with delayed discharge until hospital fees were paid. Discharge was also delayed in cases where hospital staff failed to complete required PM-JAY documentation. Follow-up visits led to recurring out-of-pocket expenses for medicines and post-operative procedures. In Gujarat, several physicians noted that the lack of PM-JAY coverage for transportation costs deterred patients from attending follow-up appointments.

## Discussion

Our study provides the first evidence on patient experiences of healthcare utilization under PM-JAY by triangulating information from multiple stakeholders—including beneficiaries, exiting patients and hospital staff—during the scheme’s early implementation. While hospitals had the required physical amenities and patient exit surveys indicated very high satisfaction with PM-JAY services, the qualitative interviews and group discussions with beneficiaries and healthcare providers revealed several areas needing procedural and service improvements. Chief among these were the need for better communication, and enhanced abilities of PM-JAY implementers—especially Ayushman Mitras and doctors—to provide empathetic and coordinated care. There were also stark differences across states: beneficiaries in Chhattisgarh, Gujarat, Kerala, Meghalaya and Tamil Nadu were more appreciative of PM-JAY services, while those in Bihar and Uttar Pradesh reported significant dissatisfaction. We further reflect upon these findings below.

We observed mixed experiences regarding the provision of **empathetic and emotional support** to PM-JAY beneficiaries. While many beneficiaries appreciated their interactions with health staff, often it was nurses or lower-level staff who respectfully engaged with them, while doctors did not have the time for this. However, doctors, especially in public facilities that received additional funds through PM-JAY reported feeling more motivated to consider patients’ needs because they now had adequate supplies and resources to do so. Beneficiaries highlighted interpersonal interactions varied based on social class and whether they were accompanied by frontline health workers, indicating the continued influence of social and structural inequities. Such disparities in responsiveness have previously been reported in both public and private facilities in India, with individuals from the lowest wealth group reporting less likely to be treated with dignity than those from the highest wealth group [40]. Additionally, caste and economic status have been shown to contribute to discriminatory practices by providers [41, 42]. Instances of non-consented care and privacy violations in our findings further underscore the need to better sensitize health providers, and the importance of incorporating formal procedures for informed consent into PM-JAY admission process.

Findings from health facilities and beneficiaries showed that the required amenities for **physical comfort of patients** were generally present. However, PM-JAY patient-specific wards were used by some hospitals to provide differentiated care. While many health providers refuted providing lower quality or different services from general wards, a deeper probing showed that having different wards sometimes enabled this.

The **communication** domain requires attention to improve patient experiences under PM-JAY, and the lack of proper awareness for communication could be observed on multiple fronts. Firstly, beneficiaries lacked access to proper communication channels about PM-JAY, and hence were inadequately or mis-informed about nearly every aspect of healthcare seeking under the scheme: eligibility and registration procedures, and services covered and hospitals where they could avail these services. This lack of awareness is corroborated by other studies from the same period [22, 43, 44], which further led to complications in coordination of care. In our sample, this was compounded by the low availability of 24/7 Ayushman Mitras in hospitals (only 16% of all hospitals had 24/7 availability, and none in Chhattisgarh and Tamil Nadu); beneficiaries often did not know who to approach for more information and were intimidated by higher-level hospital staff. Doctors observed that Ayushman Mitras had too many responsibilities to be able to adequately deal with patients. Potential solutions for this include the use of informal beneficiary navigators within and outside hospitals, as done in Indonesia, to address gaps in the formal system and increase awareness of beneficiaries [45], and in Gujarat, where centers established by a microfinance group have improved women’s community engagement in PM-JAY [46]. Many doctors voiced paying attention to communicating with patients out of a fear of complaints; PM-JAY training for doctors needs to ensure patient-responsive communication so that doctor-patient communication is not primarily motivated by complaints. Finally, all hospital staff acknowledged lacking the competencies to skillfully communicate with patients and requested training for this. We have also observed this in an earlier study, where hospital stakeholders were either inadequately or hastily trained [21]. Medical doctors suggested that formal channels of communication between them, patients and PM-JAY administrators should be established to aid interactions.

Awareness and communication issues also affected the **provision of coordination and quality of care**. While many beneficiaries reported favorable experiences, there was substantial scope for improvement. Doctors in Tamil Nadu reported that increasing awareness led to beneficiary empowerment and positive changes in health seeking behavior and adherence to treatment, while in other states information asymmetries impeded the provision of quality healthcare. Many beneficiaries reported being denied treatment because hospital staff were inadequately informed about the scheme, or preferential treatment to those socially well-connected. Beneficiaries also lamented the lack of information on the diagnoses made and benefit package availed. Whereas, doctors admitted that systemic issues between hospitals and PM-JAY administration affected service provision. Across all states, doctors reported that the perceived low reimbursement rates restricted treatment options (including the choice of drugs and consumables) for scheme patients; this was observed in another study conducted six months after PM-JAY’s launch [21]. Other areas for improvement included reducing waiting times and improving continuity of care between referral hospitals.

The **pervasiveness of OOPE** was a stark finding; while less than a third of exiting patients reported OOPE, most beneficiaries reported incurring expenditures, including informal payments for preferential treatment or to be seen by a healthcare provider. This has also been found in numerous other studies on PM-JAY [47–49]. Often, beneficiaries were neither given reasons for why they incurred OOPE, nor informed about deductions made to the coverage amount (especially when OOPE was incurred); making this information available could enhance accountability and patients’ trust in the system [50].

Finally, the findings of our study indicate a need to conduct more patient-centered research, drawing on the experiences of both patients and providers, that can feed back into the re-design and implementation of PM-JAY. Such inquiry is particularly lacking in LMICs [51]. For example, strategies like experience-based design tools have been used in the UK National Health Service to improve service delivery by capturing the experiences of patients and staff including healthcare providers, which led to the identification of facilitators like effective teamwork strategies and barriers like increased administrative burdens, among others [52]. In other high-income countries, insurers use patient-reported data for improving quality of care and value-based healthcare, including procurement and purchasing, quality assurance and strengthening the involvement of patients [10]. Based on our findings, such as the possibility to provide differentiated care in PM-JAY wards, this research must also consider pre-existing organizational and structural issues, so that new reforms do not reproduce existing systemic and social inequities, e.g., reinforcing mechanisms for segregated or poorer quality care for vulnerable or disadvantaged social groups. Further, the use of informal personal and community networks, as seen through frontline health workers in our findings, presents an opportunity to enhance empathy, emotional support and communication for beneficiaries. This can serve as a means of improving community engagement and trust in PM-JAY [50, 53]. Understanding and incorporating patients experiences of healthcare are instrumental to healthcare value co-creation [54], whereby a scheme like PM-JAY can fulfill the needs of its intended beneficiaries.

### Strengths and limitations

The strengths of our study include rich qualitative and quantitative data drawn from both community and hospital settings, from a range of respondents including doctors, PM-JAY beneficiaries in the community and patients exiting hospitals after utilizing services under PM-JAY. The data are also from a wide geographical area, drawn from 7 Indian states. There are also some limitations to our approach. Foremost, since the original study tools were designed to reflect patients and health care providers conceptualization of and experiences with scheme implementation, we did not have information on all identified initial themes relating to person/patient-centered and responsive care in the literature, as part of the first step of our analytical approach [33–36]. We were unable to examine in detail patient-centered values, preferences and shared decision-making aspects of patient experiences. Still, being one of the first studies capturing patient experiences from the PM-JAY, the study findings are critical to advance patient-centered care in the scheme specifically and PFHI in general. Due to the purposive sampling strategy, we did not have a random sample of hospitals within study districts. Further, the identification of PM-JAY beneficiaries in villages was extremely difficult and we had to rely on word of mouth or be guided by village health staff. Our study was conducted during the early implementation of PM-JAY when processes and systems to implement PM-JAY were in various stages of operationalization across states, and our findings should be interpreted in light of this. Finally, we observe stark differences between quantitative measures of satisfaction from patient exit surveys and what beneficiaries report qualitatively. This may be because of structural issues, with patients about to be discharged from hospitals more vulnerable to positive answers (potentially, also out of fear of repercussions as these were conducted in hospital premises), while those in FGDs and IDIs more at ease among their peers. Other, smaller-scale patient experience surveys from India have also reported similar high satisfaction rates [27, 28].

We also acknowledge that PM-JAY provides care within the Indian health system, and patient experiences of healthcare under PM-JAY are intertwined with systemic issues within the Indian health system. Therefore, we report on how people who are using services under the scheme experience health care within the Indian context under PM-JAY.

## Conclusion

Overall, we found patients and beneficiaries had mixed experiences of healthcare utilization under PM-JAY. While the required physical amenities in hospitals were generally available and responses from patient exit surveys indicated very high satisfaction with PM-JAY services, qualitative interviews and group discussions with beneficiaries and healthcare providers indicated several areas for improving procedures and health services. These include improvement in patient-provider communication, empathy and emotional support for patients, and coordination and quality of care. A lack of both beneficiary and health provider awareness about the scheme and inadequate communication channels and skills on the part of providers affected all aspects of utilizing healthcare under the scheme. Addressing pre-existing organizational and structural issues and using informal personal and community linkages, such as through frontline health workers, present opportunities to enhance healthcare delivery under PM-JAY.

## Electronic supplementary material

Below is the link to the electronic supplementary material.

Supplementary Material 1

## Data Availability

Proposals for data access should be directed to the Principal Investigator (MDA) manuela.deallegri@uni-heidelberg.de; data requestors will need to sign a data access agreement. The proposal for the use of data will be reviewed by a committee identified by MDA and the study funder.

## References

[1] WHO. GOAL. Target 3.8: achieve universal health coverage, including financial risk protection, access to quality essential health-care services and access to safe, effective, quality and affordable essential medicines and vaccines for all [‎poster]‎. World Heal Organ [Internet]. 3]. 2016. https://www.who.int/data/gho/data/themes/topics/indicator-groups/indicator-group-details/GHO/sdg-target-3.8-achieve-universal-health-coverage-(uhc)-including-financial-risk-protection. cited 2023 Apr 20];8. Available from.

[2] Das J, Do QT. The prices in the crises: what we are learning from 20 years of health insurance in low- and middle-income countries. J Econ Perspect [Internet]. 2023, Mar, 1;37(2):123–52. Available from: 10.1257/jep.37.2.123. cited 2023 Dec 21.

[3] Crossing the global quality chasm: improving health Care worldwide - National academies of sciences, engineering, and medicine, health and medicine division. In: Board on health Care services, board on global health, committee on improving the quality of heal [internet]. National Academies of Science; 2018. cited 2022 May 5]. Available from: https://books.google.de/books?hl=en%26lr=amp;id=_P2EDwAAQBAJ%26oi=fnd%26pg=PR1%26ots=RByobtPOoe%26sig=Pja3jkQDRqqtp38f1wx50aSgJXg%26redir_esc=y#v=onepage%26q%26f=false. 30605296

[4] Delivering quality health services DELIVERING QUALITY HEALTH SERVICES: a GLOBAL IMPERATIVE FOR UNIVERSAL HEALTH COVERAGE. http://www.wipo.int/amc/en/mediation/rules. cited 2018 May 25]; Available from. 10.1136/ejhpharm-2018-001692 PMC6452396 31157041

[5] Kruk ME, Gage AD, Arsenault C, Jordan K, Leslie HH, Roder-DeWan S, et al. High-quality health systems in the sustainable development goals era: time for a revolution. Lancet Glob Heal. 2018, Nov, 1;6(11):e1196–252.

[6] Valentine N, Darby C, Bonsel GJ. Which aspects of non-clinical quality of care are most important? Results from WHO’s general population surveys of “health systems responsiveness” in 41 countries. Soc Sci Med. 2008, May, 1;66(9):1939–50. 18313822 10.1016/j.socscimed.2007.12.002

[7] Doyle C, Lennox L, Bell D. A systematic review of evidence on the links between patient experience and clinical safety and effectiveness. BMJ Open [Internet]. 2013;3(1):1570. Available from: pmc/articles/PMC3549241/. cited 2022 May 9. 10.1136/bmjopen-2012-001570 PMC3549241 23293244

[8] Price RA, Elliott MN, Zaslavsky AM, Hays RD, Lehrman WG, Rybowski L, et al. Examining the role of patient experience surveys in measuring health care quality. Med Care Res Rev. 2014;71(5):522–54. 25027409 10.1177/1077558714541480 PMC4349195

[9] Ahmed F, Burt J, Roland M. Measuring patient experience: concepts and methods. Patient [Internet]. 2014, May, 16;7(3):235–41. Available from: https://link.springer.com/article/10.1007/s40271-014-0060-5. cited 2023 Feb 9. 10.1007/s40271-014-0060-5 24831941

[10] Neubert A, Fernandes OB, Lucevic A, Pavlova M, Gulacsi L, Baji P, et al. Understanding the use of patient-reported data by health care insurers: a scoping review. PLoS One [Internet]. 2020, Dec, 1;15(12):e0244546. Available from: https://journals.plos.org/plosone/article?id=10.1371/journal.pone.0244546. cited 2023 Feb 9. 10.1371/journal.pone.0244546 PMC7769438 33370405

[11] Yanful B, Kirubarajan A, Bhatia D, Mishra S, Allin S, Di Ruggiero E. Quality of care in the context of universal health coverage: a scoping review. Heal Res Policy Syst [Internet]. 2023, Dec, 1;21(1):21. Available from: https://health-policy-systems.biomedcentral.com/articles/10.1186/s12961-022-00957-5. cited 2023 Apr 20. 10.1186/s12961-022-00957-5 PMC10035485 36959608

[12] Carrin G, James C. Key performance indicators for the implementation of social health insurance. Appl Health Econ Health Policy [Internet]. 2005;4(1):15–22. Available from: http://link.springer.com/10.2165/00148365-200504010-00004. cited 2019 Sep 30. 10.2165/00148365-200504010-00004 16076235

[13] Ferrand YB, Siemens J, Weathers D, Fredendall LD, Choi Y, Pirrallo RG, et al. Patient satisfaction with healthcare services: a critical review [Internet]. vol. Qual Manag J. Taylor Francis. 2016;23:6–22. Available from: https://www.tandfonline.com/doi/abs/10.1080/10686967.2016.11918486. cited 2023 Feb 9.

[14] Nwanaji-Enwerem O, Bain P, Marks Z, Nwanaji-Enwerem P, Staton CA, Olufadeji A, et al. Patient satisfaction with the Nigerian National health insurance scheme two decades since establishment: a systematic review and recommendations for improvement. Afr J Prim Heal Care Fam Med. 2022;14(1):1–10. 10.4102/phcfm.v14i1.3003 PMC8831992 35144455

[15] Mohammed S, Bermejo JL, Souares A, Sauerborn R, Dong H. Assessing responsiveness of health care services within a health insurance scheme in Nigeria: users’ perspectives. BMC Health Serv Res. 2013, Dec;13:502. 24289045 10.1186/1472-6963-13-502 PMC4220628

[16] Couturier V, Srivastava S, Hidayat B, De Allegri M. Out-of-pocket expenditure and patient experience of care under-Indonesia’s national health insurance: a cross-sectional facility-based study in six provinces. Int J Health Plann Manage [Internet]. 2022, Aug, 11;n/a(n/a). Available from: 10.1002/hpm.3543. 10.1002/hpm.3543 35951490

[17] Doubova S V, Infante-Castañeda, Roder-Dewan S, Pérez-Cuevas. User experience and satisfaction with specialty consultations and surgical care in secondary and tertiary level hospitals in Mexico. BMC Health Serv Res. 2019;19(1):1–15. 31752851 10.1186/s12913-019-4706-9 PMC6873740

[18] Ravaghi H, Foroughi Z, Nemati A, Bélorgeot VD. A holistic view on implementing hospital autonomy reforms in developing countries: a systematic review. Health Policy (new Y) Plan [Internet]. 2018, Dec, 1;33(10):1118–27. Available from: https://academic.oup.com/heapol/article/33/10/1118/5245328. cited 2023 Apr 20. 10.1093/heapol/czy095 30544175

[19] Mate KS, Sifrim ZK, Chalkidou K, Cluzeau F, Cutler D, Kimball M, et al. Improving health system quality in low-and middle-income countries that are expanding health coverage: a framework for insurance. Int J Qual Heal Care. 2013;25(5):497–504. 10.1093/intqhc/mzt053 23959955

[20] Government of India. Policy Guidelines Ayushman Bharat [Internet]. [https://www.pmjay.gov.in/policy-and-guidelines. cited 2019 Sep 16]. Available from.

[21] Srivastava S, Bertone MP, Basu S, De Allegri M, Brenner S. Implementation of PM-JAY in India: a qualitative study exploring the role of competency, organizational and leadership drivers shaping early roll-out of publicly funded health insurance in three Indian states. Heal Res Policy Syst. 2023;21(1):1–14. 10.1186/s12961-023-01012-7 PMC10294452 37370159

[22] Parisi D, Srivastava S, Parmar D, Strupat C, Brenner S, Walsh C, et al. Awareness of India’s national health insurance scheme (PM-JAY): a cross-sectional study across six states. Health Policy (new Y) Plan [Internet]. 2023;38(3):289–300. Available from: 10.1093/heapol/czac106. 10.1093/heapol/czac106 PMC10019566 36478057

[23] Trivedi M, Saxena A, Shroff Z, Sharma M. Experiences and challenges in accessing hospitalization in a government-funded health insurance scheme: evidence from early implementation of Pradhan Mantri Jan Aarogya Yojana (PM-JAY) in India. PLoS One [Internet]. 2022, May, 1;17(5). Available from: https://pubmed.ncbi.nlm.nih.gov/35552557/. cited 2023 Apr 7. 10.1371/journal.pone.0266798 PMC9098065 35552557

[24] Saxena A, Trivedi M, Shroff ZC, Sharma M. Improving hospital-based processes for effective implementation of government funded health insurance schemes: evidence from early implementation of PM-JAY in India. BMC Health Serv Res [Internet]. 2022, Dec, 1;22(1):1–13. Available from: https://bmchealthservres.biomedcentral.com/articles/10.1186/s12913-021-07448-3. cited 2022 Mar 30. 10.1186/s12913-021-07448-3 PMC8760668 35031024

[25] Nandi S, Schneider H. Using an equity-based framework for evaluating publicly funded health insurance programmes as an instrument of UHC in Chhattisgarh State, India. Heal Res Policy Syst [Internet]. 2020, May, 25;18(1):50. Available from: https://health-policy-systems.biomedcentral.com/articles/10.1186/s12961-020-00555-3. cited 2021 Mar 30. 10.1186/s12961-020-00555-3 PMC7249418 32450870

[26] Ambade M, Kim R, Subramanian S V. Experience of health care utilization for inpatient and outpatient services among older adults in India. Public Heal Pract [Internet]. 2024, Dec, 1;8:100541. Available from: https://www.sciencedirect.com/science/article/pii/S2666535224000788. cited 2025 Sep 23. 10.1016/j.puhip.2024.100541 PMC11413678 39309250

[27] Oswal K, Kanodia R, Nadkar U, Kharodia N, Avhad M, Venkataramanan R, et al. Cancer patients’ experience of oncology services in Assam, India. J cancer policy [Internet]. 2021 Mar 1 [cited 2025 Sep 23], 27, 100267. Available from: https://www.sciencedirect.com/science/article/pii/S221353832030059X?casa_token=lV-UK1bgUugAAAAA:Z4_-GRixEN6hDupno59sjg_KCvAxN1KHWvDa29AuvyO2nE4295R__Dux1FB8FfArf2Kyu0H4Tw. 10.1016/j.jcpo.2020.100267 35559939

[28] Gala P, Sriram V, Kotian C, Ballala K, Vedanthan R, Perish E, et al. Perceptions of the Doctor-patient relationship among patients in a Private, secondary-level hospital in Southern India. Front Public Heal [Internet]. 2022, Apr, 6;9:768705. Available from: http://www.frontiersin.org. cited 2025 Sep 23. 10.3389/fpubh.2021.768705 PMC9019150 35463195

[29] De Allegri M, Srivastava S, Strupat C, Brenner S, Parmar D, Parisi D, et al. Mixed and multi-methods protocol to evaluate implementation processes and early effects of the pradhan mantri jan arogya yojana scheme in seven Indian states. Int J Environ Res Public Health. 2020;17(21):1–15. 10.3390/ijerph17217812 PMC7663328 33114480

[30] Bertram RM, Blase KA, Fixsen DL. Improving programs and outcomes. Implementation Frameworks Organ Change. 2016;25(4):477–87.

[31] McMahon S, Winch P. Systematic debriefing after qualitative encounters: an essential analysis step in applied qualitative research. Press; 2018. 10.1136/bmjgh-2018-000837 PMC6135453 30233833

[32] Fereday J, Muir-Cochrane E. Demonstrating rigor using thematic analysis: a hybrid approach of inductive and deductive coding and theme development: http://dx.doi.org/101177/160940690600500107. https://journals.sagepub.com/doi/10.1177/160940690600500107. cited 2021 Aug 4.

[33] Håkansson Eklund, Holmström, Kumlin T, Kaminsky E, Skoglund K, Höglander, et al. “Same same or different?” a review of reviews of person-centered and patient-centered care. Patient Educ Couns. 2019, Jan, 1;102(1):3–11. 30201221 10.1016/j.pec.2018.08.029

[34] Scholl I, Zill JM, Härter M, Dirmaier J. An integrative model of patient-centeredness - a systematic review and concept analysis. PLoS One [internet]. 2014, Sep, 17;9(9):e107828. Available from: http://www.ncbi.nlm.nih.gov/pubmed/25229640. cited 2019 Oct 4. Wu, WCH, editor.. 25229640 10.1371/journal.pone.0107828 PMC4168256

[35] Sitzia J, Wood N. Patient satisfaction: a review of issues and concepts. Soc Sci Med. 1997, Dec, 1;45(12):1829–43. 9447632 10.1016/s0277-9536(97)00128-7

[36] Batbaatar E, Dorjdagva J, Luvsannyam A, Amenta P. Conceptualisation of patient satisfaction: a systematic narrative literature review. 10.1177/1757913915594196 [Internet]. 2015, Jul, 17;135(5):243–50. Available from:https://journals.sagepub.com/doi/10.1177/1757913915594196. cited 2023 Jan 4. 10.1177/1757913915594196 26187638

[37] Glaser BG, Strauss AL, Strauss AL. The discovery of grounded theory [internet]. 2017. https://www.taylorfrancis.com/books/9781351522168. cited 2019 Jul 18]. Available from. Routledge.

[38] Patton MQ. Qualitative evaluation and research methods, 2nd ed. Thousand Oaks, CA, US: Sage Publications, Inc; 1990. p. 532 p.

[39] Rathert C, Wyrwich MD, Boren SA. Patient-centered care and outcomes: a systematic review of the literature. Med Care Res Rev. 70(4):351–79. 10.1177/1077558712465774 23169897

[40] Malhotra C, Do YK. Socio-economic disparities in health system responsiveness in India. Health Policy (new Y) Plan. 2013;28(2):197–205. 10.1093/heapol/czs051 PMC3584994 22709921

[41] Thapa R, van Teijlingen E, Regmi PR, Heaslip V. Caste exclusion and health discrimination in South Asia: a systematic review. Asia-Pac J Public Heal [Internet]. 2021, Nov, 1;33(8):828–38. Available from: https://journals.sagepub.com/doi/full/10.1177/10105395211014648. cited 2025 Apr 2. 10.1177/10105395211014648 PMC8592103 34024157

[42] Das J, Gertler PJ. Variations in practice quality in five Low-income countries: a conceptual overview. 2007, Mar, 27;26(3). 10.1377/hlthaff263.w296. 10.1377/hlthaff.26.3.w296 17389637

[43] Reddy N, B Y, K S, S M, A P, J B. Awareness and readiness of health care workers in implementing Pradhan Mantri Jan Arogya Yojana in a tertiary care hospital at Rishikesh. Nepal J Epidemiol [Internet]. 2020, Jun, 30;10(2):865–70. Available from: https://pubmed.ncbi.nlm.nih.gov/32874700/. cited 2021 Aug 5. 10.3126/nje.v10i2.27941 PMC7423403 32874700

[44] Kanore L, Satpute S. A study of awareness about Ayushyaman Bharat Yojana among low income urban Families. An exploratory study. Remarking an anal [Internet]. 2019. https://www.researchgate.net/publication/342707245_Remarking_An_Analisation_%92A_Study_of_Awareness_about_Ayushyaman_Bharat_Yojana_among_Low_Income_Urban_Families%92-An_Exploratory_Study_Sagar_Satpute. cited 2021 Aug 6];VOL-4*(ISSUE-1* (Part-1)). Available from.

[45] Raharja DP, Hanani R, Joyoadisumarta FS, Jessani NS, Mathauer I. The impact of informal patient navigation initiatives on patient empowerment and national health insurance responsiveness in Indonesia. BMJ Glob Heal [Internet]. 2022, Oct, 1 [cited 2023 Apr 13];7(Suppl 6): e009526. Available from: https://gh.bmj.com/content/7/Suppl_6/e009526. 10.1136/bmjgh-2022-009526 PMC9540845 36379590

[46] Thomas S, Sivaram S, Shroff Z, Mahal A, Desai S. ‘We are the bridge’: an implementation research study of SEWA shakti kendras to improve community engagement in publicly funded health insurance in Gujarat, India. BMJ Glob Heal [Internet]. 2022, Sep, 1 [cited 2023 Apr 13];7(Suppl 6): e008888. Available from: https://gh.bmj.com/content/7/Suppl_6/e008888. 10.1136/bmjgh-2022-008888 PMC9511541 36379589

[47] Garg S, Bebarta KK, Tripathi N. Performance of India’s national publicly funded health insurance scheme, Pradhan Mantri Jan Arogaya Yojana (PMJAY), in improving access and financial protection for hospital care: findings from household surveys in Chhattisgarh state. BMC Public Health [Internet]. 2020, Jun, 16;20(1):949. Available from: https://bmcpublichealth.biomedcentral.com/articles/10.1186/s12889-020-09107-4. cited 2020 Sep 4. 10.1186/s12889-020-09107-4 PMC7298746 32546221

[48] Prinja S, Singh MP, Aggarwal V, Rajsekar K, Gedam P, Goyal A, et al. Impact of India’s publicly financed health insurance scheme on public sector district hospitals: a health financing perspective. Lancet Reg Heal - Southeast Asia [Internet]. 2023, Feb, 1;9:100123. Available from: http://www.thelancet.com/article/S2772368222001408/fulltext. cited 2023 Jan 12. 10.1016/j.lansea.2022.100123 PMC10305929 37383034

[49] Parmar D, Strupat C, Srivastava S, Brenner S, Parisi D, Ziegler S, et al. Effects of the Indian National health insurance scheme (PM-JAY) on hospitalizations, out-of-pocket expenditures and catastrophic expenditures. Heal Syst Reform [Internet]. 2023;9(1). Available from: 10.1080/23288604.2023.2227430. 10.1080/23288604.2023.2227430 37540622

[50] Gille F, Smith S, Mays N. What is public trust in the healthcare system? A new conceptual framework developed from qualitative data in England. Soc Theory Heal [Internet]. 2021, Jan, 29;19(1):1–20. Available from: 10.1057/s41285-020-00129-x. cited 2021 Feb 13.

[51] Ibitoye BM, Garrett B, Ranger M, Stinson · Jennifer. Conducting patient-oriented research in low-income and middle-income countries: a scoping review. Patient - patient-centered outcomes res. 2022, 161 [Internet]. 16(1):19–29. Available from: https://link.springer.com/article/10.1007/s40271-022-00592-w]. 2022 Jul 22 [cited 2023 Feb 9]. 10.1007/s40271-022-00592-w 35869330

[52] Johnson A, Conroy S, Thompson D, Hassett G, Clayton A, Backhouse E. Staff experience in the NHS: a National study-an experience-based design approach. J Patient Exp [Internet]. 2022, Jan, 1. https://journals.sagepub.com/doi/10.1177/23743735221143921. cited 2023 Feb 9];9. Available from. 10.1177/23743735221143921 PMC9772966 36569263

[53] De Weger E, Van Vooren N, Luijkx KG, Baan CA, Drewes HW. Achieving successful community engagement: a rapid realist review. BMC Health Serv Res [Internet]. 2018, Apr, 13;18(1):1–18. Available from: https://bmchealthservres.biomedcentral.com/articles/10.1186/s12913-018-3090-1. cited 2023 Feb 9. 10.1186/s12913-018-3090-1 PMC5899371 29653537

[54] Lee DH. A model for designing healthcare service based on the patient experience. 10.1080/2047970020171359956 [Internet]. 2017, Jul, 3 [cited 2023 Feb 9];12(3):180–88. Available from: https://www.tandfonline.com/doi/abs/10.1080/20479700.2017.1359956.

