# Supplementary material for: “We get support now …”: a mixed methods study of patients’ experiences of healthcare under the national health insurance scheme (PM-JAY) in India
Source: BMC Health Serv Res. 2025 Nov 28;25:1552. doi: 10.1186/s12913-025-13632-6 (PMC12661883; doi:10.1186/s12913-025-13632-6)
Supplement: Supplementary file 1 — Supplementary Material 1 [file 12913_2025_13632_MOESM1_ESM.docx]

Supplementary table 1: PM-JAY Implementation in the study states

| State | Name of Scheme in the State | Name of the State Health Agency | Mode of Implementation | Date of Roll-out | Insurance Company | Insurance Support Agency (Y/N, (Number)) |
| --- | --- | --- | --- | --- | --- | --- |
|  |  |  |  |  |  |  |
| Bihar | Ayushman Bharat Pradhan Mantri Jan Arogya Yojana (PM-JAY) | Bihar Swasthaya Suraksha Samiti | Trust | 23/09/2018 | No | Yes (1) |
| Chhattisgarh* | Ayushman Bharat PM-JAY Dr. Khubchand Baghel Swasthya Bima Yojana | State Health Agency | Trust | 15/09/2018 | No | Yes (3) |
| Gujarat | Ayushman BharatPradhan Mantri Jan Arogya Yojana (PM-JAY); Mukhyamantri Amrutam & Mukhyamantri Vatsalya | Gujarat State health Protection Society | Mixed | 23/09/2018 | Yes | Yes (1) |
| Kerala | Pradhan Mantri Jan Arogya Yojana - Karunya Arogya Suraksha Paddhati (PMJAY-KASP) | State Health Agency, Kerala | Trust | 01/04/2019 | No | Yes (1) |
| Meghalaya | Megha Health Insurance Scheme (MHIS) Pradhan Mantri Jan Arogya Yojana (PM-JAY) | State Nodal Agency Meghalaya | Insurance | 23/09/2018 | Yes | Yes (1) |
| Tamil Nadu | Pradhan Mantri Jan Arogya Yojana-Chief Minister's Comprehensive Health Insurance Scheme (PMJAY-CMCHIS) | Tamil Nadu Health System Project (TNHSP) | Mixed | 23/09/2018 | Yes | Yes (2) |
| Uttar Pradesh | Ayushman Bharat Pradhan Mantri Jan Arogya Yojana (PM-JAY) and Mukhiya Mantri Jan Arogya Abhiyan (MMJAA) | State Agency for Comprehensive Health and Integrated Services (SACHIS) | Trust | 23/09/2018 | No | Yes (4) |

*As of July, 2020, Chhattisgarh transitioned from a mixed mode of implementation to a trust mode.

Source: Adapted from <https://pmjay.gov.in/states/states_glance>
